# Percutaneous afferent lymphatic vessel sclerotherapy for postoperative lymphatic leakage after previous ineffective therapeutic transpedal lymphangiography

**DOI:** 10.1186/s41747-020-00188-9

**Published:** 2020-11-02

**Authors:** F. Pan, M. Loos, T. D. Do, G. M. Richter, H. U. Kauczor, T. Hackert, C. M. Sommer

**Affiliations:** 1 Clinic for Diagnostic and Interventional Radiology, University Hospital Heidelberg, INF 110, 69120 Heidelberg, Germany; 2 Department of Radiology, Union Hospital, Tongji Medical College, Huazhong University of Science and Technology, Wuhan, China; 3 Department of General, Visceral and Transplantation Surgery, University Hospital Heidelberg, Heidelberg, Germany; 4 Clinic for Diagnostic and Interventional Radiology, Stuttgart Clinics, Katharinenhospital, Kriegsbergstrasse 60, 70174 Stuttgart, Germany

**Keywords:** Chyle, Lymphatic vessels, Lymphography, Postoperative complications, Sclerotherapy

## Abstract

**Background:**

To demonstrate the efficacy of percutaneous computed tomography (CT)-guided afferent lymphatic vessel sclerotherapy (ALVS) in the treatment of postoperative lymphatic leakage (LL) after ineffective therapeutic transpedal lymphangiography (TL).

**Methods:**

A retrospective review in this institute involving 201 patients was conducted from May 2011 to September 2018. Patients diagnosed with postoperative LL undergoing ineffective therapeutical TL before the performance of percutaneous CT-guided ALVS were involved. Technical success and clinical success of TL and ALVS were established. The technical success and efficacy of ALVS in the treatment of postoperative LL after ineffective therapeutic TL were assessed. The clinical success rate of ALVS is also assessed, and the complications are reviewed.

**Results:**

In total, nine patients were involved including three patients (33.3%) presented with chylothorax, three patients (33.3%) presented with inguinal lymphatic fistula/lymphocele, and three patients (33.3%) presented with lymphatic fistula in the thigh; 27 ± 18 days (mean ± standard deviation) after surgery, therapeutic TL was successfully performed and showed definite afferent lymphatic vessel and leakage site in all the patients. Due to clinical failure after TLs, the following ALVS was performed with a mean interval of 12 ± 8 days after TL. The technical success rate was 9/9 (100.0%, 95% confidence interval [CI] 63.1–100.0%). An average of 2.7 ± 1.3 mL 95% ethanol as sclerosant agent was injected during the procedure. The clinical success was observed in 8 of the 9 patients (88.9%, 95% CI 51.8–99.7%) with a time between ALVS and the LL cure of 8 ± 6 days. No complications were reported.

**Conclusions:**

Our results showed the role of percutaneous CT-guided ALVS as a safe, feasible, and effective salvage treatment for postoperative LL after ineffective TL.

## Key points

- Lymphatic leakage is a severe complication after surgery.
- Conventional transpedal lymphangiography is a therapeutic approach with a failure rate of 30%.
- Percutaneous computed tomography-guided afferent lymphatic vessel sclerotherapy is an effective treatment for refractory postoperative lymphatic leakage.

## Background

Postoperative lymphatic leakage (LL) is a severe complication that could result in a fatal event [1–3]. In the management of patients with postoperative LL in different locations, the conventional transpedal lymphangiography (TL) is an established diagnostic and therapeutic approach with an efficacy rate of about 70% for curing LL [4, 5]. However, for the patients who underwent ineffective TL, the exploration of salvage therapy with minimal invasion is still meaningful.

Because of the high risk of the surgical revision, several alternative interventional treatments have been developed based on TL, such as percutaneous thoracic duct embolisation (TDE), thoracic duct disruption (TDD), transnodal embolisation (TNE), direct pooling-leakage embolisation, and afferent lymphatic vessel sclerotherapy (ALVS) [3, 6, 7]. These interventional treatments are minimally invasive, safe, and relatively simple to be performed.

In the ALVS procedure, a small-sized needle was used to puncture close to the afferent lymphatic vessels that were responsible for the leakage. Then, the sclerosant was injected to destroy the lymphatic vessels and prevent leakage. In year 2014, Kortes et al. [6] reported that their use of percutaneous computed tomography (CT)-guided ALVS successfully cured 7 of 10 patients (70%) with LL. However, half of the patients underwent TL with simultaneous ALVS. Hence, it is still questionable to estimate the real efficacy of ALVS for postoperative LL while excluding the therapeutic influence of TL.

In this study, the aim was to demonstrate whether this percutaneous CT-guided ALVS could become an effective salvage treatment after ineffective TL for postoperative LL.

## Methods

### Definitions and criteria

Before performing this retrospective study, different definitions and criteria were set up based on the published literature. Diagnosis criteria for LL were milky fluid leakage with triglycerides above 110 mg/dL and/or positive detection of the chylomicron [8, 9]. The technical success of TL was defined as uneventful cannulation of the lymphatic vessels with injected contrast opacification of the lymphatics extending beyond the site of the identified extravasation [10, 11]. The technical success of percutaneous CT-guided ALVS was defined as the distribution of the sclerosant around the target lymphatic vessel which was assessed under the CT scan [6]. The clinical success of TL or percutaneous CT-guided ALVS was defined as complete cessation of lymph leakage after the procedure or a gradual decrease resulting in the withdrawal of the drainage tube in 3 weeks and lack of the need for other treatment, such as conservative medical nutrition therapy, *e.g.,* a fat-free or low-fat oral supplement with medium-chain triglycerides (MCTs), enteral feeding and/or parenteral nutrition with a high percentage of MCTs etc., and surgical ligation. Further, the clinical failure was defined as no change or an increase of the LL in the follow-up, with the necessity of a conservative treatment or any other additional treatment (*e.g.,* surgical revision, radiotherapy) [5, 12, 13].

### Study population

The institutional digital databases including the General Electric picture archiving and communication system and centricity radiology information system databases (General Electric Medical Systems, Buckinghamshire, UK) were retrospectively retrieved for the period from May 2011 to September 2018. The inclusion criteria were as follows: (1) patients diagnosed with postoperative LL, (2) technically successful TL performed for the postoperative LL with the intention to cure the LL, (3) percutaneous CT-guided ALVS performed after the identification of clinical failure of the previous TL.

The electronic data of 201 patients who were diagnosed with postoperative LL and underwent therapeutical TL was retrieved by two independent radiologists (C.M.S. and F.P., with more than 10 and 8 years of experience in interventional radiology, respectively).

### Therapeutic TL

The therapeutic TL was performed for the patients with clinically diagnosed lymphatic leakage from the lower extremity, iliac, abdominal para-aortic lymphatics, or thoracic duct [14]. Informed consent was obtained from all patients. The details of the TL technique have previously been described [6, 15]. A period of 20–40 min after the injection of 1 mL of 1:3 mixture of methylene blue dye and local anesthetic between the interdigital spaces of the foot, an accessible lymphatic vessel on the dorsum of the foot was examined. Then, a small incision was made and the lymphatic vessel was carefully dissected free and catheterised with the use of a 26-gauge intravenous cannula (BD Vasculon Plus; BD, Heidelberg, Germany). Afterwards, the iodinated oil (Lipiodol, Guerbet Germany, Sulzbach, Germany) was manually injected with the velocity of approximately 0.5–2.0 mL/min to opacify the lymphatic vessels under fluoroscopy (Artis Zee, Siemens Healthineers, Erlangen, Germany). The maximal volume of the iodinated oil was less than 20 mL according to the instructions for use and in order to prevent fatal pulmonary embolisation [4]. The needles were removed, and the wounds were sutured after the accomplishment of the injection. After the iodinated oil reached the leakage level or above as observed on the TL images, the post-TL CT scan (Somatom Definition DS; Siemens Healthineers, Erlangen, Germany) was performed for identifying the site of LL and the afferent lymphatic vessel with the assistance of the TL images [6, 16]. All the procedures were performed by the same team of interventional radiologists with experience of over 20 years in lymphangiography, lymphatic sclerotherapy, and embolisation.

### Indication and contraindications to percutaneous CT-guided ALVS

The indication of percutaneous CT-guided ALVS was the definite identification of the leakage site and the afferent lymphatic vessels feeding the leakage on the post-TL CT images [6]. The contraindications included the proximity of the target area to the important vessels or nerves and anticoagulant/antithrombotic medication [6].

### Percutaneous CT-guided ALVS

**Fig. 1:**
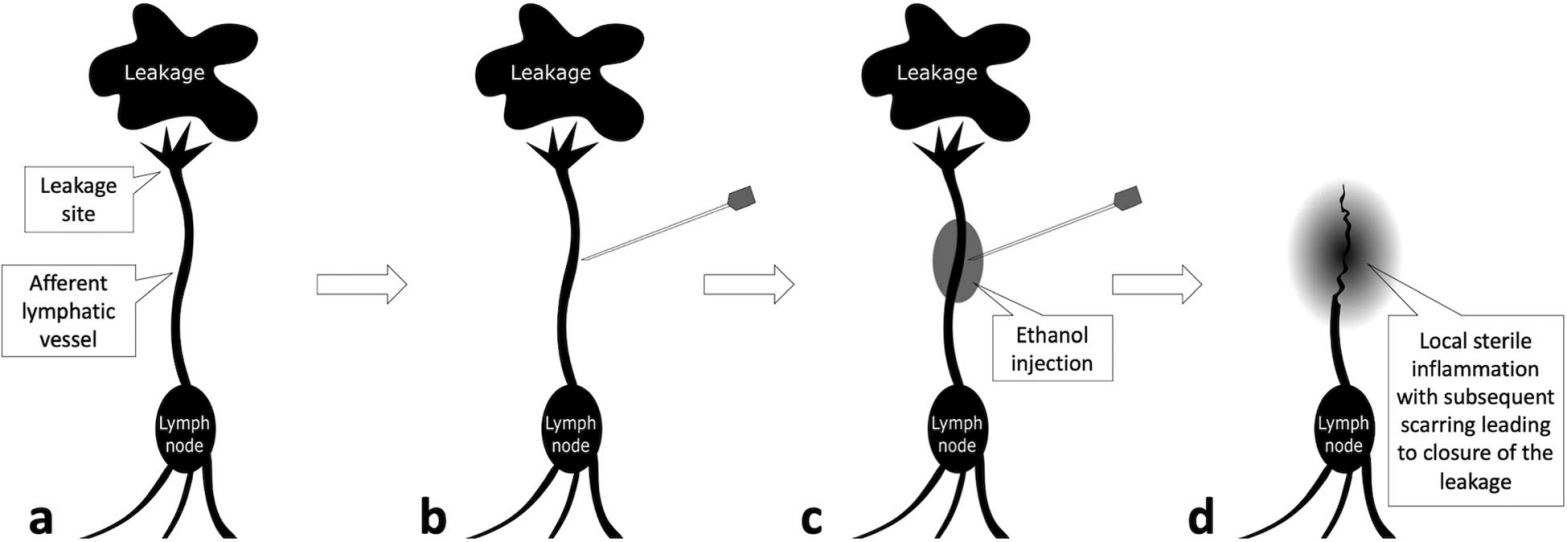
The ALVS treatment. **a** The definite LL site and the afferent lymphatic vessel as the target for the following ALVS treatment should be identified using TL and CT. **b** In the ALVS procedure, the needle was used to puncture as close as possible to the afferent lymphatic vessel. **c** After the confirmation of the ideal distribution of the prior injected contrast, 95% ethanol as sclerosant was injected. **d** Afterwards, the local sterile inflammation led to the local scarring and subsequent obliteration of the afferent lymphatic vessel. Then, the leakage ceased. *ALVS* Afferent lymphatic vessel sclerotherapy, *CT* Computed tomography, *LL* Lymphatic leakage, *TL* Transpedal lymphangiography

The percutaneous CT-guided ALVS was performed after the clinically failed TL treatment. The technique was described before and an illustration is shown in Fig. 1 [6]. Informed consent was obtained from all patients. The procedure was performed under local anesthesia, and 7.5 mg piritramide was intravenously injected before the procedure. Nasal-cannula oxygen therapy was performed with a velocity of 2 to 3 L/min during the procedure. The noninvasive multifunctional electrocardiogram, pulse oximetry, and blood pressure monitor were routinely performed during the procedure. The preoperative CT was performed after the placement of a radiopaque optical marker on the skin of the patient, and the target lymphatic vessel was confirmed with the assistance of the previous CT images. The CT parameters were set as follows: single-energy technique, tube voltage 120 kVp, effective tube current-time product from 16 to 383 mAs. Images were reconstructed as along the axial plane with a slice thickness of 3 mm and increment of 3 mm. All reconstructions were performed in a medium soft-tissue kernel (B31f; Siemens Medical Solutions, Siemens, Forchheim, Germany).

Then, the ideal access point and route were meticulously chosen to avoid unnecessary injury to any other organ or structure. After administration of local anesthesia (Xylocaine 1%, Astra Zeneca, Wedel, Germany), the 21- or 22-gauge Chiba needle (15 or 20 cm in length) or the 21-gauge hypodermic needle (5 cm in length) was used to puncture with the tip placed as close as possible to the afferent lymphatic vessels, depending on the depth of the target lymphatic vessels. Then, approximately 1–4 mL of a 1:9 mixture of iomeprol 300 mgI/mL (Iomeron 300, Bracco Imaging Deutschland, Konstanz, Germany) and local anesthetic was injected to observe whether the contrast dispersed around the target lymphatic vessel without distribution into any other important tissue or structure. Afterwards, 95% ethanol (B. Braun Melsungen, Melsungen, Germany) as sclerosant agent was injected with a volume lower than 5 mL [6]. After the sclerotherapy, a CT scan was performed again to assess possible intraprocedure complications. All the procedures were performed by a team of interventional radiologists as mentioned above.

After the procedure, patients were transferred to the ward and asked for a 2-h bed rest with compression of the puncture site as well as noninvasive multifunctional electrocardiogram, pulse oximetry, and blood pressure monitor. If the cardiorespiratory manner was stable after 2 h; then, the patient was allowed to leave the bed. Analgesia was performed depending on the clinical situation.

### Follow-up

After ALVS therapy, the daily chyle drainage output was recorded, and intermittent CT/x-ray was carried out. After discharge, the patients were asked for a regular recheck at the outpatient department and/or after hospitalisation every 1 to 3 months lasting for half a year.

### Study goals

After the retrieval of patient involvement consent, patient information was collected, including age, sex, diagnosis, surgery, location of the LL, daily chyle/lymph output, the clinical outcome of ALVS, and complications, etc. The study goals of the ALVS included technical success rate, clinical success rate, the interval between the ALVS and the cure of LL, and the complications. The procedure-related complications were collected and classified into minor and major complications in accordance with the criteria of the Society of Interventional Radiology [17].

### Statistical analysis

Statistical analyses were performed using the IBM SPSS Statistics Software (version 24; IBM, New York, USA). Quantitative data were presented as mean ± standard deviation, while the counting data were presented as count and percentage of the total. Because this was not a comparative study, only descriptive analysis was used. For the rates of technical and clinical success, the 95% confidence interval (CI) was calculated according the Clopper-Pearson test.

## Results

### Basic characteristics

From the database review, no patient was excluded from ALVS therapy due to contraindications. In total, there were 9 patients made up of 6 men and 3 women aged 63 ± 10 years (range 49–81 years). Malignancy was the major etiology, in that 7 of 9 patients (77.8%) were diagnosed with different types of carcinoma or sarcoma. Only 2 of 9 patients (22.2%) were diagnosed with benign diseases. After the surgeries, 3 patients (33.3%) presented with chylothorax, 3 patients (33.3%) presented with inguinal lymphatic fistula, and 3 patients (33.3%) presented with a lymphatic fistula in the thigh. The mean daily chyle/lymph output was 541 ± 545 mL (range 100–1,500 mL). All patients could not be cured by prior conservative medical nutrition therapy (a fat-free or low-fat oral supplement with MCTs, enteral feeding, and/or parenteral nutrition with a high percentage of MCTs). Furthermore, 3 of 9 patients (33.3%) underwent surgical revisions, and 1 of 9 patients (11.1%) underwent three transdrainage doxycycline instillations (100 mg) before the performance of TL, but the leakage was still consistent without any alleviation [18].

Therapeutic TLs were performed with a mean time of 27 ± 18 days (range 13–70 days) after surgery. An average of 11.8 ± 5.0 mL iodinated oil (range 5.0–20.0 mL) was injected during the procedure. The post-TL CT showed a definite afferent lymphatic vessel and a leakage site in all the patients. In the follow-up, the clinical failure of TLs was identified in all enrolled patients. Thus, the percutaneous ALVS as a salvage treatment was performed. Details are shown in Tables 1 and 2.

**Table 1:** Patient characteristics and treatment details

Patient number	Other treatments to cure LL except for conservative therapy before TL	Diagnosis	Surgery	LL location	Daily chyle/lymph output (mL/day)	Time interval between surgery and TL (days)	Volume of iodinated oil injected in TL (mL)	Interval between TL and ALVS (days)	Volume of 95% ethanol injected in ALVS (mL)	Clinical outcomes of ALVS	Salvage treatment after clinically failed ALVS	Interval between ALVS and the cure of LL (days)
**1**	None	Esophagus carcinoma	Thoracic-abdominal esophagus resection + lymphadenectomy	Chylothorax	1,100	22	16.0	8	4.0	Clinical failure	Yes: surgical revision	13
**2**	None	Erosive esophagitis induced esophageal stenosis	Thoracic-abdominal esophagus resection + lymphadenectomy	Chylothorax	1,130	15	15.0	3	4.0	Clinical success	No	8
**3**	None	Thoracic aortic aneurysm (dislocation of the grafts)	Descending aorta replacement	Chylothorax	1,500	14	15.0	8	2.0	Clinical success	No	7
**4**	Surgical revision	Cutaneous Merkel cell carcinoma	Inguinal lymphadenectomy	Inguinal lymphatic fistula	200	28	9.0	5	5.0	Clinical success	No	8
**5**	Surgical revision	Penile carcinoma	Inguinal lymphadenectomy	Inguinal lymphatic fistula	340	24	20.0	13	2.0	Clinical success	No	7
**6**	Surgical revision	Malignant melanoma	Inguinal lymphadenectomy	Inguinal lymphatic fistula	100	70	5.0	10	1.0	Clinical success	No	1
**7**	None	Chondrosarcoma	Bone tumour resection	Lymphatic fistula at the thigh	300	13	6.0	11	2.0	Clinical success	No	20
**8**	Doxycycline instillation (100 mg/3 times)	Malignant melanoma	Inguinal lymphadenectomy	Lymphatic fistula at the thigh	100	18	10.0	18	2.0	Clinical success	No	3
**9**	None	Malignant melanoma	Inguinal lymphadenectomy	Lymphatic fistula at the thigh	100	36	10.0	28	2.0	Clinical success	No	1

*ALVS* Afferent lymphatic vessel sclerotherapy, *LL* Lymphatic leakage, *TL* Transpedal lymphangiography

**Table 2:** Patient characteristics and treatment outcome

		***N*** = 9
Sex	Male	6 (66.7%)
	Female	3 (33.3%)
Diagnosis	Malignant melanoma	3 (33.3%)
	Esophagus carcinoma	1 (11.1%)
	Thoracic aortic aneurysm	1 (11.1%)
	Penile carcinoma	1 (11.1%)
	Erosive esophagitis induced esophageal stenosis	1 (11.1%)
	Cutaneous Merkel cell carcinoma	1 (11.1%)
	Chondrosarcoma	1 (11.1%)
Surgery	Inguinal lymphadenectomy	5 (55.6%)
	Thoracic-abdominal esophagus resection and lymphadenectomy	2 (22.2%)
	Descending aorta replacement	1 (11.1%)
	Bone tumour resection	1 (11.1%)
Location of the lymphatic leakage	Chylothorax	3 (33.3%)
	Inguinal lymphatic fistula	3 (33.3%)
	Lymphatic fistula at the thigh	3 (33.3%)
Other treatment to cure LL except for the conservative therapy before TL	None	5 (55.6%)
	Surgical revision	3 (33.3%)
	Doxycycline instillation	1 (11.1%)
Clinical outcomes of the ALVS treatment	Clinical success	8 (88.9%)
	Clinical failure	1 (11.1%)
Salvage treatment after the clinical failure of the ALVS	Surgical revision	1 (11.1%)
Age (years)		63 ± 10 (49–81)
Daily chyle/lymph output (mL/d)		541 ± 545 (100–1,500)
Interval between surgery and TL (days)		27 ± 18 (13–70)
Volume of iodised oil injected in TL (mL)		11.8 ± 5.0 (5.0–20.0)
Interval between TL and ALVS (days)		12 ± 8 (3–28)
Volume of 95% ethanol injected in ALVS (mL)		2.7 ± 1.3 (1.0–5.0)
Interval between ALVS and the cure of LL (days)		8 ± 6 (1–20)

*ALVS* Afferent lymphatic vessel sclerotherapy, *LL* Lymphatic leakage, *TL* Transpedal lymphangiography

### Study goals

**Fig. 2:**
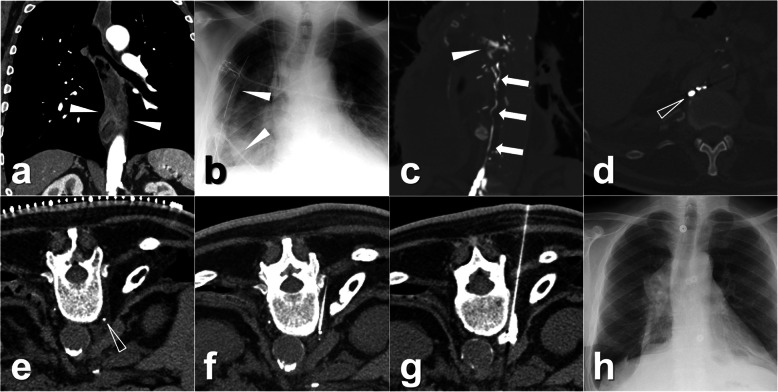
Images from a patient who was diagnosed with erosive esophagitis-induced esophageal stenosis. **a** Contrast-enhanced CT showed an irregular thickness of the distal esophageal wall (white arrowheads). Then the thoracic-abdominal esophagus resection plus lymphadenectomy was performed. After the surgery, the right chylothorax appeared with the average daily output of 1,130 mL. **b** The two drainage catheters (white arrowheads) were also observed at the ideal locations in the chest radiogram. Due to the ineffective total parenteral nutrition with a high percentage of MCTs, TL was performed, and 15.0 mL of iodinated oil was injected. **c** Eight hours later, CT scan showed a definite rupture (white arrowhead) of the thoracic duct (white arrows) with the extravasation to the right pleural cavity. **d** The distal cisterna chyli (white hollow arrowhead) was regarded as the target afferent lymphatic vessel. Because of the gradual increase of the thoracic drainage after TL, ALVS was performed 3 days later as a salvage. **e** In the preoperative CT scan, a small amount of residual iodinated oil could still be seen at the cisterna chyli (white hollow arrowhead). **f** Then, the planned puncture was achieved, and the tip was close to the cisterna chyli. **g** Afterwards, 1 mL if contrast agent was injected and showed an ideal distribution around the cisterna chyli. After that, 4.0 mL 95% ethanol was injected. After the procedure, the chylothorax output gradually reduced until the drainage could be removed on day 8 after ALVS. **h** Eleven days after ALVS, the chest radiogram showed no obvious recurrence of the chylothorax. The patient was discharged on the next day. *ALVS* Afferent lymphatic vessel sclerotherapy, *CT* Computed tomography, *MCTs* Medium-chain triglycerides, *TL* Transpedal lymphangiography

**Fig. 3:**
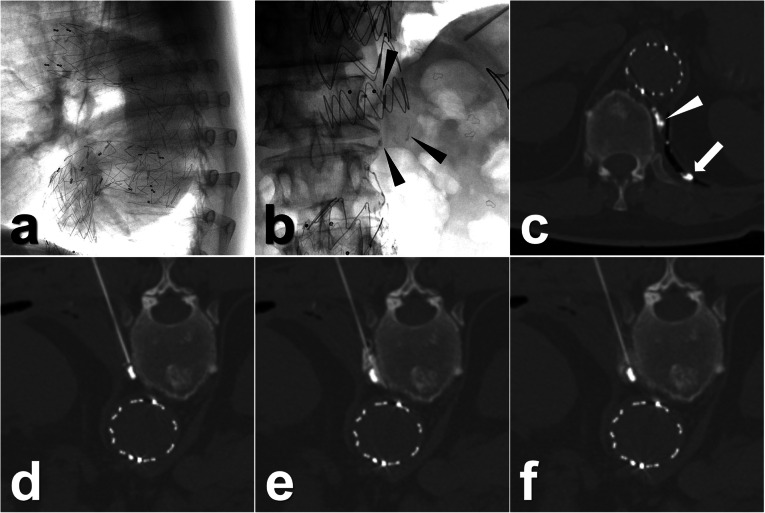
Images from a patient who underwent endovascular aneurysm repair procedures for the progressive thoracic aortic aneurysm 4 years before. **a** In the recent follow-up, the dislocation of the thoracic grafts was found. Then, aortic replacement surgery was performed. After the surgery, the left chylothorax was diagnosed with a daily drainage output of around 1,500 mL/day. **b** In the TL, a small amount of iodinated oil extravasation was observed (black arrowheads) near the left crural diaphragm. **c** Also, CT definitely showed the extravasation from the ruptured thoracic duct (white arrowhead) into the pleural cavity (white arrow). Eight days later, the percutaneous ALVS was performed owing to there being no obvious regression of the drainage. **d** During the procedure, the needle puncture was close to the afferent thoracic duct. **e** Then, 1 mL of contrast fluid was injected that showed an ideal distribution around the thoracic duct. **f** Afterwards, 2.0 mL 95% ethanol was injected; the final CT scan showed a good distribution of the prior injected contrast fluid surrounding the target lymphatic vessel. From then on, the chylothorax output gradually reduced and completely ceased after 1 week. Then, the drainage was removed and the patient was discharged on the next day. *ALVS* Afferent lymphatic vessel sclerotherapy, *CT* Computed tomography, *TL* Transpedal lymphangiography

**Fig. 4:**
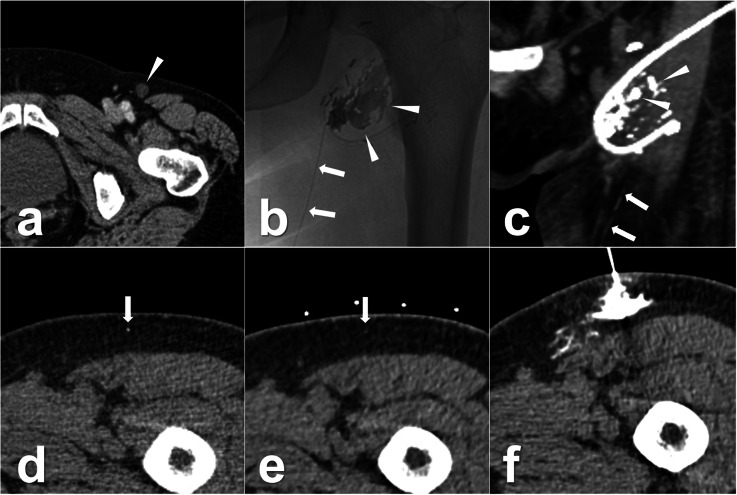
Images from a patient who underwent a melanoma resection on the left leg 4 months before. **a** A suspicious relapse of one left inguinal lymph node (white arrowhead). Then, a left inguinal lymphadenectomy was performed, and the pathological result confirmed the lymph node metastasis. After this surgery, the continuous LL was observed with an average output of 100 mL/day. Owing to the ineffective surgical revision, the TL was performed and definite iodinated oil extravasation (white arrowheads) from one lymphatic vessel (white arrows) was found using both fluoroscopy (**b**) and a sequential CT scan, obtaining coronal (**c**) and axial (**d**) reconstructions. Ten days after TL, percutaneous ALVS was performed due to there being no regression of the drainage. **e** In the preoperative CT scan, the afferent lymphatic vessel (white arrowhead) could still be identified. **f** Afterwards, a successful puncture was achieved, and 1 mL of contrast fluid was injected that showed an ideal distribution around the target lymphatic vessel. Then, 1.0 mL 95% ethanol was injected. One day later, the leakage ceased completely, and the drainage was removed. The patient was discharged on the same day. *ALVS* Afferent lymphatic vessel sclerotherapy, *CT* Computed tomography, *LL* Lymphatic leakage; *TL* Transpedal lymphangiography

**Fig. 5:**
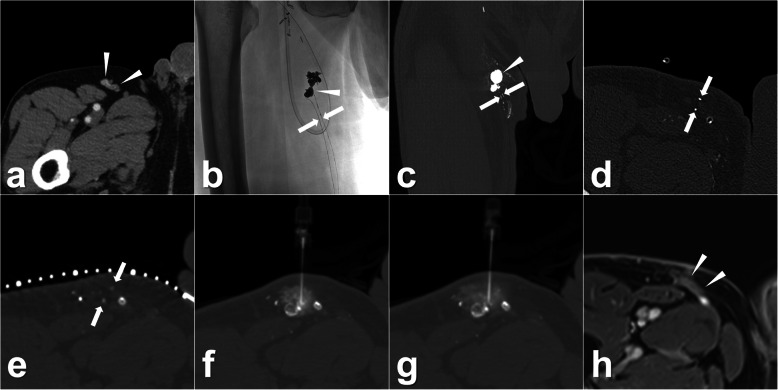
Images from a patient who underwent a melanoma resection 1 year before. **a** Contrast-enhanced CT showed a suspicious metastatic lymph node (white arrowheads) at the right proximal thigh. Hence, a right inguinal lymphadenectomy was performed, and the postoperative pathological result confirmed the metastasis of the lymph node. Because of the postoperative LL, the therapeutic TL was performed showing definite iodinated oil extravasation (white arrowhead) from two lymphatic vessels (white arrows) using both fluoroscopy (**b**) and a CT scan, obtaining coronal (**c**) and axial (**d**) reconstructions. Twenty-eight days after TL, percutaneous ALVS was performed owing to the continuous leakage. **e** The two target afferent lymphatic vessels (white arrows) close to the leakage could still be identified. **f** During the puncture, the needle tip was located between the two afferent lymphatic vessels, and 1 mL of contrast fluid was injected that showed an ideal distribution covering both the target lymphatic vessels. **g** After injection of 2.0 mL of 95% ethanol, a CT scan was performed which showed a nebulous dispersal of the contrast completely covering the two target lymphatic vessels. One day later, the leakage ceased and the drainage could be removed. The patient was discharged on the same day. **h** Around 3 months later, magnetic resonance imaging showed no tumour recurrence and fluid accumulation. A mild enhancement (white arrowheads) at the region of sclerotherapy could be observed which indicated clinically inapparent scarring after ALVS. In the further follow-up for 1 year, no relapse of the tumour or lymphatic leakage was found. *ALVS* Afferent lymphatic vessel sclerotherapy, *CT* Computed tomography, *LL* Lymphatic leakage, *TL* Transpedal lymphangiography

The interval between the TL and percutaneous ALVS was 12 ± 8 days (range, 3–28 days). In the procedures, the afferent lymphatic vessels could still be differentiated in all patients with the assistance of previous post-TL CT images. The technical success of TLs was 9/9 (100.0%, 95% CI 63.1–100.0%). An average of 2.7 ± 1.3 mL (range, 1.0–5.0 mL) 95% ethanol was injected. The clinical success was observed in 8 of 9 patients (88.9%, 95% CI 51.8–99.7%), the time between ALVS and LL cure being 8 ± 6 days (range 1–20 days). For instance, four cases with LL at different locations including chylothorax, inguinal lymphatic fistula, and lymphatic fistula in the thigh are demonstrated in Figs. 2, 3, 4, and 5. In the follow-up lasting for half a year, no LL recurrence was observed. No minor or major complication was reported. In the other three patients with clinically failed treatment, other salvage treatments were performed, such as surgical revision, doxycycline instillation, and radiotherapy. Details are shown in Tables 1 and 2.

## Discussion

In this study, percutaneous CT-guided ALVS was successfully performed in nine patients. As a salvage therapy after the clinically failed TL, it achieved a clinical success rate of 88.9%, and no ALVS-related complication was reported.

Basically, the interventional treatments are classified into two types: (1) those embolising the responsible lymphatic vessels (*e.g.*, TDE, TNE) and (2) those that destroy them (*e.g.*, ALVS, TDD). Nevertheless, the definite site of LL should be confirmed using TLs as the premise in the majority of cases [1, 6, 7, 13].

Indeed, there are different limitations for most interventional modalities. For instance, for TDE and afferent lymphatic vessel embolisation, finding the accessible feeding lymphatic vessel is essential [1, 3, 7]. If the feeding lymphatic vessel is very small, it will be infeasible to perform these procedures. Instead, TNE is a better alternative. Nevertheless, the lymph node should be very close to the extravasation; otherwise, it will be difficult for the embolising agent to embolise the feeding lymphatic vessels completely [19]. Notwithstanding, the direct pooling-leakage embolisation/sclerotherapy cannot be performed on ruptured lymphoceles or direct lymphorrhea manifestation such as chylous ascites [13].

Compared with other techniques, the results of the present study show that ALVS has a broader application for treating the postoperative LL at different locations in clinical practice with a high technical success rate (see Figs. 2, 3, 4, and 5). As the objective of ALVS in this study was to induce local sterile inflammation leading to subsequent obliteration of the afferent lymphatic vessel (see Fig. 1), a precise puncture into the lymphatic vessel is not very necessary [6]. TDE, which was the most classic embolisation technique for chylothorax, only had a technical success rate of about 50 to 60% [1, 20, 21]. When the catheterisation fails, then TDD could become the salvage [1, 3, 20]. However, it is very difficult to identify whether the responsible lymphatic vessels were destroyed in multiple punctures. But in ALVS, the prior contrast injection enabled the identification of the following sclerosant distribution. Thus, it is more reliable to completely sclerotise the responsible lymphatic vessels.

Considering the good efficacy of the conventional TL, only the patients undergoing clinically failed TL were involved in the present study. As a result, the clinical success rate of ALVS reached 88.9%. By comparison, TDD was reported with a clinical success rate in the range of 41.7 to 72.2% [1, 3, 20]. TDE showed a clinical efficiency rate in the range of 84.6 to 90.5% [1, 3, 20]. Further, TNE also showed an efficiency rate of about 80% [13, 22]. It would seem that the embolisation of the afferent lymphatic vessels had a better efficiency than ALVS. But all these data came from the patients undergoing successful TL with simultaneous interventions. Nevertheless, the time between ALVS and the cure using LL in patients with clinical success was less than 1 week, which is similar to the results when using other techniques [13].

Although ethanol has a very strong destruction capability with respect to tissue, no ALVS-related complication was found in this cohort. To prevent ectopic destruction, the prior contrast injection was very important in order to show the potential distribution of the follow-on ethanol injection. Even after the ethanol sclerotisation, the nebulous opacity of the contrast in the final re-check of the CT scan could also be observed, indicating the actual ethanol distribution (see Figs. 3 and 5).

This study has some limitations. First, the samples were very limited in number, which meant that no statistical comparison could be performed to find the prognostic risk factors. Second, the study cohort was heterogeneous with the different sites of postoperative LL. Although the treating principles of ALVS were the same for the different sites of LL, this study did not determine whether there was any difference in efficiency for different LL. Thus, a more specifically observational or controlled study will still be necessary in future studies.

In conclusion, our results showed the role of percutaneous CT-guided ALVS as a safe, feasible, and effective salvage treatment for postoperative LL after ineffective TL. The further exploration of this technique would be significant regarding its clinical application.

## Data Availability

All data generated or analysed during this study are included in this published article.

## Abbreviations

ALVS: Afferent lymphatic vessel sclerotherapy
CT: Computed tomography
LL: Lymphatic leakage
MCTs: Medium-chain triglycerides
TDD: Thoracic duct disruption
TDE: Thoracic duct embolisation
TL: Transpedal lymphangiography
TNE: Transnodal embolisation
